# Application of artificial liver in immune-related liver injury induced by immune checkpoint inhibitor: Case reports and review of the literature

**DOI:** 10.3389/fimmu.2022.1001823

**Published:** 2022-09-02

**Authors:** Xuewei Li, Lina Ji, Xiaofang Li, Dong Sun, Wenhui Yang

**Affiliations:** ^1^ Department of Biochemistry and Molecular Biology, Shanxi Key Laboratory of Birth Defect and Cell Regeneration, Shanxi Medical University, Taiyuan, China; ^2^ Department of Digestive Oncology, Cancer Center, Shanxi Bethune Hospital, Shanxi Academy of Medical Sciences, Tongji Shanxi Hospital, Third Hospital of Shanxi Medical University, Taiyuan, China; ^3^ Department of Radiology, Shanxi Bethune Hospital, Shanxi Academy of Medical Sciences, Tongji Shanxi Hospital, Third Hospital of Shanxi Medical University, Taiyuan, China

**Keywords:** immune checkpoint inhibitors (ICIs), artificial liver, immune-related adverse events (irAEs), immune-mediated hepatotoxicity (IMH), immune

## Abstract

The use of immune checkpoint inhibitors (ICIs) can improve survival of patients with malignant tumors, however, the ICI treatment is associated with unpredictable toxicity as immune-related adverse effects (irAEs). Here we report two cases of metastatic malignant gastrointestinal tumors where severe immune-mediated hepatotoxicity (IMH) developed, characterized by liver failure, after the ICI therapy. Through a strong immunosuppressive treatment and a non-biological artificial liver and supportive treatment, the liver function was restored in both cases, and the anti-tumor treatment effect was guaranteed. These results showed that the non-biological artificial liver could be capable of improve prognosis during the ICI therapy.

## Background

Immune checkpoint inhibitors (ICIs) have been shown to be effective in prolonging the survival of patients with various cancers, and a variety of ICIs are currently being developed and applied as monotherapy or combination therapy. However, in clinical applications by modulating immune response, ICIs may be associated with irAEs, potentially affecting diverse organs, including liver. These irAEs have different degrees, and can sometimes be fatal and life-threatening. ICIs-induced immune-mediated hepatotoxicity (IMH) is often manifested as asymptomatic elevation of alanine aminotransferase (ALT) and/or aspartate aminotransferase (AST), with or without increased bilirubin. It may also be accompanied by non-specific symptoms such as fever, fatigue, and decreased appetite. Elevated bilirubin can cause yellow skin and sclera, yellow urine, etc. However, in rare cases, IMH can even cause acute liver failure (1).

For the treatment of IMH, various guidelines recommend the use of glucocorticoids as the main treatment drugs. For patients without any contraindication to hormones, the active hormone therapy is recommended. For patients with rapid disease progression or liver failure, plasma exchange may be an optional treatment (2).

As one of the effective methods for the treatment of liver failure, the non-biological artificial liver removes various harmful substances from liver, supplements essential substances to liver, and improves the internal environment within liver. It can temporarily compensate a part of the function of failed liver and create physiological conditions for the regeneration of liver cells, and the recovery of liver function was conducted by an *in vitro* mechanical, physicochemical and biological device. Currently, the following modes are commonly used in clinical practice for liver failure: plasma exchange (PE), double filtration plasmapheresis (DFPP), double plasma molecular absorb system (DPMAS), and plasma diafiltration (PDF). For the present study, we treated two patients with liver failure induced by ICIs by application of the artificial liver, which effectively restored liver function in both cases. Our results showed a remarkable clinical effect by the application of artificial liver.

## Case presentation

### Case 1

A 54-year-old man was admitted to our hospital on August 11th, 2021 due to yellow staining of skin and sclera for 18 days and fever for 3 days. On April 22th, 2021, the color doppler ultrasound revealed pancreatic space-occupying, following this, relevant examinations were performed to confirm the diagnose of pancreatic head cancer, gastric wall invasion, and abdominal lymph node metastasis. From June 2 to June 23th, 2021, the systemic chemotherapy combined with immunotherapy was given to this patient, more specifically: nab-paclitaxel 200mg d1, 8+ Seggio Capsules 60mg bid d1-14, sintilimab 200mg d1/3w were injected for this combined treatment. His liver function was normal before treatment. On July 5th, 2021, the patient developed a scattered rash all over the body, which was diagnosed as immune-related dermatitis. This symptom of the patient was improved after a hormone therapy. On July 25th, 2021, fatigue, anorexia, and yellowing of the skin and sclera were observed in the patient. The patient had fever on August 8th, with a body temperature of up to 39°C and without cough, expectoration, dysuria, urgency, abdominal pain, and diarrhea. The antibiotic and glucocorticoid (2 mg/kg/d) treatment for the patient lasted 3 days, resulting in a disatisfactory treatment effect. Afterwards, the patient was admitted to the hospital for further diagnosis and treatment. He had no history of hypertension, diabetes, heart disease, blood transfusion, and drug allergy. Physical examination: the sclera and skin of the whole body were severe yellow staining, scattered erythema and papules, and no liver palm and spider nevus were observed.; the breath sounds of both lungs were clear, with the absence of wet or dry sounds; the heart sounded strong, with a regular rthythm of a heart rate of 68 beats/min; the abdomen was soft, with the absence of tenderness and rebound tenderness; the liver and spleen were not under the ribs; no percussion pain in the liver area was observed, and mobility dullness was negative; no edema was noted on either leg.

Laboratory examinations showed the following results: Whole blood cell analysis: WBC:11.2×109/L, NEUT#:8.68×109/L, PLT:43×109/L; Coagulation: PT:29.3s, INR:2.74, PTA:26%, APTT:35.7s; Blood biochemical examination: ALT:1736.4IU/L, AST:800IU/L, TBil:167μmol/L, DBil:98.8μmol/L, D/T:0.59, γ-GT:256.7IU/L, ALP:102.2IU/L; HBsAg, HBeAg, HBcAg, HBsAb, HBeAb, HBcAb, Hepatitis A antibody, Hepatitis C antibody and Hepatitis E antibody IgM were all negative. AFP: 5.8ng/mL; autoantibodies were negative; Immunoglobulin IgG10.8g/L, IgA 3.84g/L, IgM0.73g/L. Abdominal MRI (September 10th, 2021): cystic mass in the neck of the pancreas with nodules on the right wall. Liver puncture was not performed for the patient due to coagulation dysfunction. Final diagnosis: Immune-related liver injury induced by ICIs, grade 4, with a subacute liver failure, early. The diagnosis basis of this patient is shown in **Table 1** (3).

**Table 1:** Grading and management principles of immune-mediated liver injury induced by ICI.

Grading	Management
G1: Asymptomatic (AST or ALT >ULN to 3.0 × ULN and/or total bilirubin >ULN to 1.5 × ULN)	Continue ICPi with close monitoring; consider alternate etiologies.Consider monitoring labs 1-2 times weekly.Manage with supportive care for symptom control.Hold ICPi temporarily.Patients should be advised to stop unnecessary medications and any known hepatotoxic drugs. Temporarily hold other potentially hepatotoxic oncologic agents.
G2: Asymptomatic (AST or ALT> 3.0 to ≤ 5 × ULN and/or total bilirubin > 1.5 to ≤ 3x ULN)	For grade 2 hepatic toxicity, may administer steroid (0.5-1 mg/kg/d prednisone) or equivalent if no improvement is seen after 3-5 days.Increase frequency of monitoring to every 3 days. If inadequate improvement after 3 days, consider adding mycophenolate mofetil. May initiate steroid taper when symptoms improve to ≤ G1 and may resume ICPi treatment when steroid ≤ 10 mg/d. Taper over at least 1 month. Consider hepatology consult for G2 and above. May resume if recover to ≤ G1 on prednisone ≤ 10 mg/d.Follow G2 recommendations as listed, with the following additions for G3: Consider permanently discontinuing ICPi if asymptomatic; permanently discontinue if symptomatic.Immediately start steroid 1-2 mg/kg methylprednisolone or equivalents.
G3: AST or ALT 5-20 × ULN and/or total bilirubin 3-10 × ULN, OR symptomatic liver dysfunction; fibrosis by biopsy, compensated cirrhosis; and reactivation of chronic hepatitis	If steroid refractory, consider liver biopsy to rule out NASH, tumor, cholestatic variants, other drug-related hepatic inflammation, infection, or other autoimmune entity and consider adding azathioprine^b^ or mycophenolate^c^ if infectious cause is ruled out.Labs daily or every other day: consider inpatient monitoring for patients with AST/ALT > 8 × ULN and/or elevated total bilirubin 3 × > ULN.If no improvement is achieved with steroid or for patients on ICPi therapy combined with a novel agent, with standard CTX, or with targeted therapy, refer to hepatologist for further pathologic evaluation of hepatitis.Steroid taper can be attempted around 4-6 weeks when ≤ G1, re-escalate if needed, optimal duration unclear.Consider transfer to tertiary care facility if necessary.
G4: AST or ALT > 20 × ULN and/or total bilirubin > 10 × ULN OR decompensated liver function (eg, ascites, coagulopathy, encephalopathy and coma)	Follow G3 recommendations as listed, with the following additions for G4:Administer 2 mg/kg/d methylprednisolone equivalents

**Figure 1:**
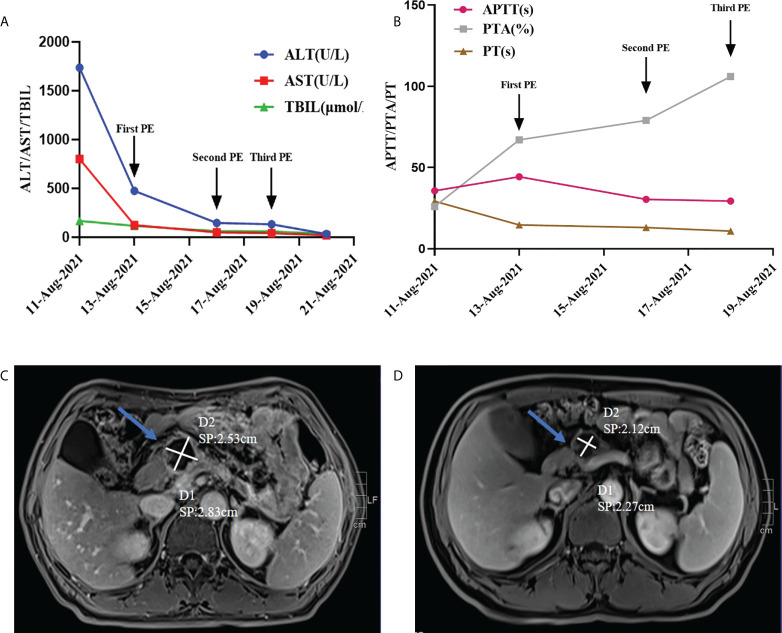
**(A, B)** Changes of various indicators before and after artificial liver treatment; **(C)** On September 10, 2021, the abdominal MRI of the pancreatic neck was about 2.5*3.04cm in size (indicated by the arrow); **(D)** On December 9, 2021, the abdominal MRI of the pancreatic neck occupies a space of about 2.16*2.37cm (indicated by the arrow).

Then, Sindilizumab was stopped immediately, intravenous magnesium isoglycyrrhizinate 200mg once a day, AdenosylMethionine 1g once a day and glucocorticoid (2mg/kg/d) were given. And on August 13th, 16th, and 18th, the single-level plasma exchange (PE) treatment was performed respecitively, and the dose of each treatment was the same, namely 2000ml fresh frozen plasma. Liver function and coagulation indicators were improved significantly after this treatment (see **Figures 1A, B**). Afterwards, sintilimab was discontinued, and systemic chemotherapy was used for 3 cycles, the liver function was normal during this period of treatment. Repeat abdominal MRI on December 9th: the size of cystic mass in the neck of the pancreas was smaller than that observed on September 10th, 2021 (see **Figures 1C, D**).

### Case 2

A 64-year-old woman was admitted to our hospital on December 3th, 2021 due to the “diagnose of liver metastatic adenocarcinoma for more than 3 months and fatigue for 1 week”. In early August 2021, the patient had been admitted to our hospital because of abdominal pain and low back pain. PET-CT(Positron emission tomography-computed tomography 2021/8/13): 1.Multiple hypermetabolic nodules and masses in the liver; low density shadow of portal vein, considering tumor thrombus; Multiple lymph node metastasis in mediastinum, hepatogastric space, hilar region and retroperitoneum; Multiple bone metastasis. 2.A hypermetabolic mass in the hilar region is considered to be malignant. Further pathological immunohistochemical (IHC) results showed that AE1/AE3 (+), Hepatocyte (partial +), AFP (-), GPC-3 (focal +), HSP70 (+), GS (-), Arginase-1 (focal +), IMP3 (diffuse +), CK7 (minority +), CK20 (+), CEA (-), CDX-2 (-), SATB2 (+), Villin (+), GATA-3 (-), TTF-1 (-), Napsin A (-), p53 (80% +). Ki67 (30% +). Based on the results of imaging examination and pathological immunohistochemistry, the patient was diagnosed as intrahepatic cholangiocarcinoma with multiple metastases after MDT multi-disciplinary consultation. cTNM staging: stage IV. From August 16th to November 8th, paclitaxel (albumin-bound) combined with Seggio and pembrolizumab was given to the patient for 5 cycles. Her liver function was normal before treatment. At the end of November 2021, the patient developed fatigue, anorexia, and abdominal distension, without the symptom of abdominal pain, fever, nausea, and vomiting. She was admitted to the hospital for further diagnosis and treatment. The patient has a history of hepatitis B, but no history of hypertension, diabetes, heart disease, blood transfusion, and drug allergy. Her physical examination revealed the following: The sclera and skin of the whole body were normal, no liver palm and spider nevus were seen; the breath sounds of both lungs were clear, with the absence of dry and wet sounds; the heart sounded strong, with a regular rhythm of a heart rate of 62 beats/min; the abdomen was soft, without tenderness and rebound tenderness; the liver was 4 transverse fingers below the right ribs, and the mobility dullness was negative; no edema was noted on either leg.

Laboratory examinations showed the following results: Whole blood cell analysis (2021-12-4): WBC:2.8×109/L, NEUT#:1.61×109/L, PLT:57×109/L; Coagulation (2021-12-4): PT:14.6s, INR:1.34, PTA:66%; Blood biochemical examination (2021-12-4): ALT:774IU/L, AST:1113.5IU/L, TBil:26.6μmol/L, DBil:13.5mol/L, γ-GT:160.6IU/L, ALP:229.5IU/L;ICI was stopped immediately, intravenous magnesium isoglycyrrhizinate 200mg, reduced glutathione 1.8g once a day to protect the liver.Other examination (2021-12-4): HBsAg(+)、HBeAg(+)、HBcAg(-)、HBsAb(-)、HBeAb(+)、HBcAb(+), HBV DNA:<100IU/mL, Hepatitis A antibody, Hepatitis C antibody and Hepatitis E antibody IgM were all negative.AFP:3.5ng/mL; autoantibodies were negative; Immunoglobulin IgG9.5g/L、IgA 2.58g/L、IgM1.2g/L. Liver biopsy was recommended, which was rejected by the family. (2021-12-10):ALT(228.4IU/L) and AST(749.3IU/L) were lower than before,TBil(119.3 umol/L) and γ-GT(188IU/L) were higher than before, and she gradually developed yellow skin and sclera, dark urine, extreme fatigue, abdominal distension, and nausea. Considering immune-mediated hepatotoxicity, glucocorticoid(1mg/Kg/d) was given (12/10-12/20), Combined with oral mycophenolate mofetil(MMF) 0.5g twice a day (12/18-12/20). Blood biochemical examination (2021-12-20): ALT: 842.6IU/L, AST: 1249.7IU/L, TBil: 249.4μmol/L, DBil: 93.8mol/L. Coagulation (2021-12-25): PT: 26.2s, INR: 2.34, PTA: 30%, APTT: 39.1s. Abdominal CT (2021-12-26): hepatic hilar space occupying and liver metastasis of the patient was improved compared with that in the previous examination. Liver cirrhosis, enlarged spleen, widened portal vein were observed in the patient during this period of examination. Final diagnosis: Immune-related liver injury induced by ICIs, grade 4. with a subacute liver failure at an early stage. Diagnosis was confirmed based on the same results as previous ones.

**Figure 2:**
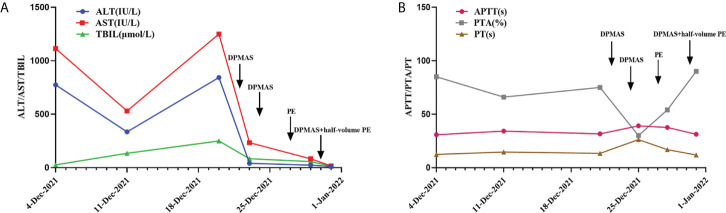
**(A, B)** Changes of various indicators before and after artificial liver treatment.

**Figure 3:**
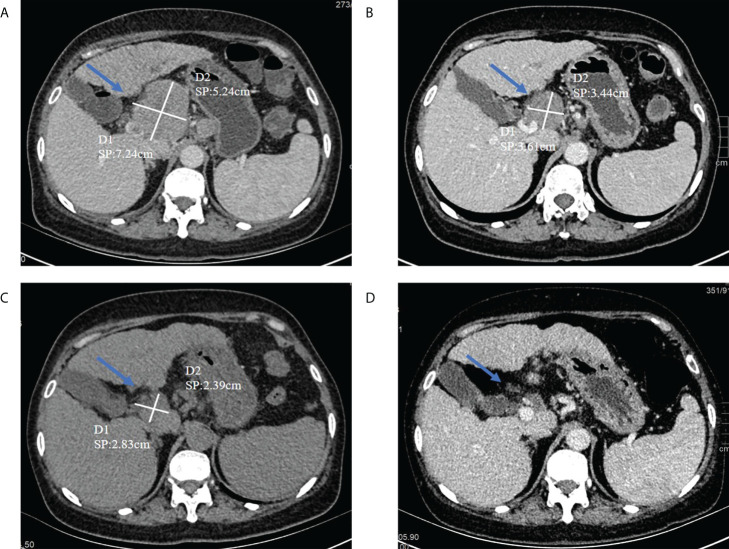
**(A)** On August 6, 2021, abdominal CT (before treatment) had a tumor in the hepatic hilum area, with a size of about 5.24*7.64cm (indicated by the arrow); **(B)** The tumor in the hepatic hilum area on September 25, 2021, about 3.44*3.61cm in size (indicated by the arrow); **(C)** On January 20, 2022, abdominal CT (after treatment) had a tumor in the hepatic hilum area, with a size of about 2.39*2.83cm(indicated by the arrow); **(D)** On Apirl 23, abdominal CT showed that the tumor had basically disappeare.

Subsequently, from December 22th to 24th, she was given a double plasma molecular adsorption therapy. On December 27th, she underwent single-plex plasma exchange, replacing 2000ml of fresh frozen plasma. On December 30th, double plasma molecular adsorption combined with half-volume plasma exchange therapy was given to her, and the amount of plasma exchanged was 1000ml (see **Figure 3**). After this treatment, her liver function returned to normal (see **Figures 2A, B**). Subsequently, the re-examination showed that her liver lesions had shrunk significantly (see **Figures 3A–D**).

## Discussion

With the widespread use of ICIs, irAEs have increasingly attracted the attention of clinicians. Among them, IMH induced by ICIs can even lead to liver failure or death, and in severe cases it can threaten the life of patients. According to clinical reports, the rate of occurrence of ICIs-induced IMH is approx 3-10% (4–6). The pharmoco-vigilance database of World Health Organization (WHO) showed that among 613 fatal adverse events caused by ICIs this year, 20.2% (124/613) of patients died of IMH (7). These factors indicate that the risk of death caused by IMH should be paid more attention.

The mechanism of IMH occurrence is not fully understood. The liver has evolved high immune tolerance due to continuous exposure to foreign antigens, and blockade of immune checkpoints may lead to abnormal immune activation, affecting the liver of patients. At present, it has been found in human specimens and animal models that the occurrence of IMH is largely caused by the activation of CTL. CD8^+^ T-predominant lymphocytic infiltration has been observed in both human and mouse livers treated with ICIs and elevated transaminases (8–10). In addition, various CD4^+^ T helper cells and Treg cells are also involved in the liver injury of IMH (11, 12). It is speculated that the possible mechanism is that ICIs induce lymphocyte activation and mediate hepatocyte death by expressing death ligands, exocytosing granules or producing various cytokines such as IFN-γ and TNF (10). In addition, secondary activation of the innate immune system has also been found to be involved in immunosuppressive-induced liver injury (11). It has been reported that hepatocyte injury and cell death in IMH may be caused by the crosstalk between innate and adaptive immunity (13). However, its specific mechanism has not been fully elucidated.

IMH has been reported to occur 6 to 12 weeks after the first ICI dose (14), but may also occur after longer periods of treatment. In this case 1, the patient developed multiple rashes all over the body after using ICIs for 12 days, which considered skin damage from ICIs. After hormonal treatment, the patient improved, with fatigue, anorexia, yellow skin, significantly elevated liver enzymes, and coagulation dysfunction in 53 days. And excluded viral hepatitis, alcoholic liver disease, non-alcoholic fatty liver disease, autoimmune liver disease, cholestatic disease, genetic metabolic disease and other liver diseases. No liver tumor lesions or biliary obstruction were found by imaging examination, and the RUCAM scale judged causality with a score of 8 (highly likely). After ICIs treatment, the patient developed skin and liver damage successively, and multiple irAEs have been observed in clinical practice (15). Nearly half of IMH patients have other types of irAEs (4), so the combination of irAEs is more helpful for the diagnosis of IMH. In this case, the diagnosis of immune-related liver injury and hepatocyte injury induced by ICIs was established.

In case 2, the patient developed fatigue and anorexia after using ICIs for 14 weeks. Laboratory tests showed a significant increase in transaminase, followed by a rapid decline in transaminase, progressive deepening of jaundice, and abnormal coagulation (PTA decreased to 30%). Although combined with a history of hepatitis B, the long-term antiviral treatment effect is good, and the HBV DNA detection is within the normal range. Liver diseases such as alcoholic liver disease, non-alcoholic fatty liver disease, autoimmune liver disease, cholestatic disease, and genetic metabolic disease were excluded. Imaging examination of the liver tumor was smaller than before, and no biliary obstruction was found. So, the patient was diagnosed with immune-related liver injury induced by ICIs.

At present, major guidelines (16, 17) all use the liver toxicity grading standard (according to the level of transaminase and bilirubin) in the common terminologycriteria for adverse events (CTCAE) to guide the treatment of IMH. Glucocorticoids are the main treatment drugs recommended by the guidelines, which have the effect of suppressing the immune response. It can improve the immune damage of target organs, stabilize the lysosomal membrane, reduce the non-specificity of portal area and capillary bile ducts, prevent the production of antigen-antibody complexes through anti-endotoxin effect, prevent further damage to liver function, and gradually improve liver function. If there is no response to hormone therapy for 3 days, the addition of mycophenolate can be considered. The current guidelines do not make clear recommendations for third-line immunosuppressive therapy. Tacrolimus and antithymocyte globulin (16) can be added. Considering the issue of liver toxicity, infliximab is not recommended. Two patients had rapid disease progression after liver injury, and they were diagnosed with subacute liver failure. After adequate glucocorticoid, liver protection, jaundice, nutritional support and other comprehensive treatment, the effect is not good.

Non-biological artificial liver supportive therapy has become an important treatment method for liver failure and has been widely used in the clinical applications, and its clinical efficacy has been demonstrated in different studies (18). It was reported that plasma exchange was used to treat acute liver failure caused by yellow phosphorus poisoning, the overall survival rate of patients reached 79.06% (19).Despite low overall incidence rate of IMH, fulminant liver failure(0.1% to 0.2%) can occur sporadically (20). Artificial liver therapy such as plasma exchange can remove a large amount of toxins, simultaneously infuse fresh normal plasma and supply diverse nutritious factors such as albumin and coagulation factors, which effectively compensates the liver detoxification and synthesis function and provides optimal internal environment for liver repair. During and/or after artificial liver therapy, the liver enzymes can return to a normal status, the level of bilirubin decreases, and the coagulation function is improved. These results showed that this therapy is effective, which not only saves the patient’s life, but also form a clinical basis for follow-up therapeutic intervention.

In the present study, the patient in case 1 experienced recovered liver function following artificial liver therapy and continued anti-tumor treatment. The re-examination showed that the volume of tumor lesion was shrunk by more than 30% according to the evaluation criteria for solid tumors (RECIST 1.1). The overall treatment result was evaluated as partial remission, and the patient successfully completed conversion therapy and is currently undergoing a surgical treatment. The patient in case 2 was diagnosed as advanced hepatic malignant tumor. After artificial liver therapy, the liver function of this patient was improved. The re-examination result showed that the shrunk volume of tumor lesion in this patient was more than 80%. The overall evaluation of treatment was partial remission. Unfortunately, the results of liver biopsy were not obtained due to the significant abnormal blood coagulation and high risk of liver puncture bleeding in case 1, and the refusal of the family members of case 2, which needs to be further improved.

## Conclusion

In summary, liver failure caused by immune checkpoint inhibitors can be treated with artificial liver therapy that can significantly improve the liver function and ensure therapeutic effectiveness and safety during anti-tumor treatment.

## Data availability statement

The original contributions presented in the study are included in the article/Supplementary Material. Further inquiries can be directed to the corresponding author.

## Ethics statement

Written informed consent for the publication of any identifiable images or data was obtained from the patient.

## Author contributions

XuL, LJ, XiL, DS and WY: study concept and design the overall study. XuL, LJ and XL: analyzed and interpreted the patient data. XL and WY: drafted the manuscript. All authors contributed to the article and approved the submitted version.

## Funding

This case report was supported by grants from the National Natural Science Foundation of China (No. 82002619), the Key Medical Research Projects of Shanxi Province (No. 2020XM55), and the Talent Introduction Scientific Research Start-up Fund of Shanxi Bethune Hospital (No. 2020RC006).

## Acknowledgments

We thank the patients for their permission to publish this case report and for their friendly cooperation with providing necessary data.

## Conflict of interest

The authors declare that the research was conducted in the absence of any commercial or financial relationships that could be construed as a potential conflict of interest.

## Publisher’s note

All claims expressed in this article are solely those of the authors and do not necessarily represent those of their affiliated organizations, or those of the publisher, the editors and the reviewers. Any product that may be evaluated in this article, or claim that may be made by its manufacturer, is not guaranteed or endorsed by the publisher.
